# Trust tangles: Who do kids trust? Unraveling young children’s attitudes in the gossip mill

**DOI:** 10.3389/fpsyg.2026.1852349

**Published:** 2026-07-22

**Authors:** Songsong Wang, Xinru Wang, Weixian Liang, Lei Dai, Jinyan Song, Xiaobo Gao, Junting Huang, Xinyi Yu, Hongmin Bao, Qiang Zhou, She Zheng

**Affiliations:** 1 Department of Psychology, Wenzhou Medical University, Wenzhou, China; 2 School of Foreign Language Studies, Wenzhou Medical University, Wenzhou, China

**Keywords:** gossip, interpersonal trust, moral evaluation, social preference, young children

## Abstract

**Objective:**

As an important channel of social-information transmission, gossip enables individuals to acquire reputational information indirectly and to guide partner selection. This study systematically investigates the cognitive development from social evaluation to trust-based decision-making in children. Through two experiments, we examine how young children evaluate different roles in gossip scenarios and how those evaluations influence subsequent trust decisions.

**Methods:**

Study 1 examined how 141 children (aged 5–7) integrated factors such as gossip valence, emotional cues, and information transmission frequency to form moral evaluations and social preferences toward gossip spreaders. Study 2 employed a Trust Game paradigm with 108 children aged 5–6 to evaluate how a spreader’s reputation and social relationships affect trust judgments and behavioral decisions in contexts involving negative gossip.

**Results:**

Findings indicate task-dependent age differences among 5- and 6-year-olds in gossip contexts. Specifically, for moral evaluations of spreaders, 6-year-olds attended more to information valence (positive vs. negative testimony) than 5-year-olds, who relied primarily on transmission frequency. In Trust Games, both age groups adjust trust based on role-specific reputation and relational information.

**Conclusion:**

These findings underscore that children’ reputation-based trust judgments exhibit early stability, while their integrated moral evaluations of motives become more refined with age.

## Highlights

- Young children are already capable of integrating complex social cues to evaluate roles and engage in Trust Decision-Making within gossip ecosystems.
- In gossip contexts, 5- and 6-year-olds exhibit age-related differences in complex moral judgments but show consistency in intuitive Trust Decision-Making.
- Age 6 represents a critical developmental turning point for children integrating motivation into moral evaluations of gossip, providing a precise window for phased social education.

## Introduction

Gossip, defined as evaluative information about absent third parties, allows individuals to gain behind-the-scenes insights into others’ behaviors and characteristics (Foster, 2004). As a ubiquitous form of social communication, it functions as an efficient channel for social learning (Hartung et al., 2019). Importantly, gossip is not confined to adults but is observable in early developmental stages during childhood (Engelmann et al., 2016).

As children enter formal schooling, their expanding peer networks require the independent management of social interactions. To adapt, children must learn about others not only through direct contact but also through indirect information acquisition (Over and Carpenter, 2012). *Gossip transmission*—defined as evaluative third-party information about an absent individual—serves as a key mechanism for this indirect learning (Foster, 2004). As a critical social tool, gossip transmission allows children to indirectly assess their peers’ behaviors and traits, influencing their social judgments and relationship decisions (Dores Cruz et al., 2021; Shinohara et al., 2024). Building on the theoretical frameworks of indirect reciprocity and partner selection, gossip serves as a mechanism for the exchange of information regarding the past behaviors of others, circumventing the need for direct interaction (Nowak, 2006). This process aid individuals in making informed decisions about partner selection within cooperative settings, thereby protecting them from exploitation by unreliable partners and enhancing the potential for mutual benefits (Gross et al., 2024). Nonetheless, while gossip facilitates the assessment of others’ reputations, it simultaneously subjects individuals to scrutiny. The dissemination of negative information about an individual through gossip can result in adverse social repercussions for that person (Nieper et al., 2022). Given the distinctive role of gossip in sustaining human cooperative networks, it is imperative for individuals to cultivate skills that enable them to select reliable partners for interaction and learning in environments rich with gossip, while simultaneously avoiding deception by dishonest actors (Boehm, 2012). This raises the question: How do young children, whose social cognition is still in the developmental stage, discern and process such intricate information? How do they assess the intentions and reliability of those who disseminate gossip, and how do they identify genuinely trustworthy individuals within these contexts?

Previous research indicates that children begin to attend to reputation as early as age 3 and increasingly express evaluations of others through gossip. By approximately age 5, children are capable of conveying third-party information to peers via prosocial gossip, suggesting that young children are already experimenting with gossip as a social tool (Zhang and Tang, 2021).

Children not only recognize the social significance of gossip but also differentiate communicators based on the valence of the information they convey. Specifically, children hold a strong positive bias. They give more positive moral evaluations and show greater preference toward positive gossip spreaders, while judging negative gossip transmitters unfavorably and keeping them at a distance (Caivano and Talwar, 2021; Tang et al., 2023). Even in situations without factual support, children prioritize accepting positive gossip while dismissing negative gossip; this valence-based selective preference remains consistent across early childhood (Tang et al., 2023). Notably, children are more sensitive to negative gossip, questioning communicators’ credibility and forming negative moral judgments when facts conflict. Positive gossip fits inherent expectations and rarely faces scrutiny (Wan et al., 2025).

Apart from content valence and affective bias, exposure frequency also affects children’s judgments. Repeated exposure boosts familiarity and preference via the mere exposure effect and shapes their evaluations (Becker and Taylor, 2023). Therefore, the focus of our research in Experiment 1 is that children evaluate gossip spreaders based primarily on the social valence of the information they communicate. We put out the following hypothesis:

Hypothesis 1: Children will evaluate positive gossip spreaders more favorably than negative gossip spreaders, both morally and socially.

We further explored whether contextual cues, including context valence (i.e., whether the context was positive or negative) and transmission frequency, modulate children’s evaluations of gossip spreaders.

However, realistic social engagement necessitates not only evaluating others but also making consequential decisions based on those evaluations—particularly trust decisions about whether to take risks, rely on information, or act on a communicator’s assertions (Thielmann and Hilbig, 2015). Gossip is not always beneficial; it can be self-serving and may reflect the spreader’s reaction to perceived misbehavior against authority persons (Vaish et al., 2011). Negative gossip is a particularly insidious form of relational aggression in which teachers or peers discreetly communicate damaging or misleading information about a student in order to harm that student’s reputation (Ellwardt et al., 2012). Such negative gossip correlates with social exclusion, peer rejection, and a higher risk of psychological distress, including elevated suicidal thoughts among targeted (Wang et al., 2020). Accordingly, Experiment 2 focuses on negative gossip scenarios that produce high-conflict decision contexts in order to investigate factors that shape children’s trust.

Previous studies indicate that besides gossip valence, communicators’ reputation, intentions and interpersonal relationships also influence children’s trust (Köymen and Engelmann, 2022). Children trust those with benevolent intentions more and reduce trust when detecting deceptive motives (Liu et al., 2013). With advancing cognitive development, school-age children can recognize conflicting intentions rooted in group interests and correspondingly adjust their trust strategies toward in-group and out-group members (Hermes et al., 2018; Ye et al., 2026). Children’s gossip evaluations are affected by social bonds. They oppose gossip spreaders more strongly when targets are close friends than ordinary peers (Caivano and Talwar, 2021). If personal experience conflicts with gossip, children adjust trust based on the spreader’s reputation (Wan et al., 2026).

Experiment 2 further examined that children’s trust in gossip depends not only on gossip valence, but also on characteristics of the communicator. We propose the following hypotheses:

Hypothesis 2: Children will show reduced trust toward negative gossip, particularly when the spreader has a poor reputation.

Hypothesis 3: Children will place greater trust in gossip communicated by socially familiar peers than by strangers.

We additionally explored developmental differences across age groups.

### Study 1

In happy/unhappy scenarios, the study examines young children’s moral evaluations, social preferences, and attitude assessments toward gossip spreaders across varying frequencies and positive/negative testimonies. It specifically looks into who children might choose as friends, how they perceive the spreaders’ behavioral characteristics, and how they feel about these gossip disseminators generally.

## Method

### Participants

Using G*Power 3.1.9.7, an *a priori* power analysis with an effect size of *f* = 0.25, *α* = 0.05, and power (1 − *β*) = 0.80 indicated a minimum sample of 128 children. We recruited 141 children (age range: 5–7 years) from two kindergartens (M_age_ = 6.11 years, SD = 0.74). Table 1 shows the descriptive statistics of participants in Study 1, and detailed group assignments are provided in Supplementary Table S1. The study was approved by the relevant institutional ethics committee. Written parental consent and verbal assent from each child were obtained before testing.

**Table 1:** Demographic information of the Study 1 sample by age group.

Age group (years)	*N*	Mean age (years)	SD (years)	Male	Female
5	31	5.00	0.00	16	15
6	63	6.00	0.00	34	29
7	47	7.00	0.00	23	24
Total	141	6.11	0.74	73	68

*N*, number of participants; SD, standard deviation.

### Procedure

Following Caivano et al. (2020), we prepared short videos that illustrated gossip propagation in everyday contexts. Experimental programming and stimulus presentation were implemented in PsychoPy 3.0. Each video had three distinct actors, differentiated by fixed costume colors and assigned to specific testimonial roles: the gossip spreader (yellow; positive testimony), the target of gossip (red; neutral testimony), and the gossip receiver (blue; negative testimony). In each scenario, each actor delivered three statements, with the order of the statements randomized. Participants completed three independent trials. Each trial consisted of five stages: practice, introduction, emotional-context, gossip moral-rating, and feedback. To control for potential gender effects, we presented gender-matched versions of the stimuli and counterbalanced scenario content across participants. Children were told they could choose a mystery gift box after completing all trials. The key constructs in Study 1 were moral evaluation, attitude evaluation, social preference, emotional context, testimony valence, and transmission frequency. Definitions, operationalizations, example manipulations, and scoring procedures for these constructs are summarized in Supplementary Table S2.

During the practice phase, to ensure children understood and could use the rating scales, the experimenter first explained the basic concept of scoring using smiley faces (representing “good”) and frowny faces (representing “bad”). Next, children practiced rating their preference for five snacks and cartoon characters using a five-point preference scale (1 = strongly dislike, 5 = strongly like) to familiarize themselves with the procedure. Children could respond using gestures or by selecting pictures. All participants must demonstrate mastery of the rating scale before proceeding to the main experiment.

During the introduction phase, the computer screen presented the three task characters. To ensure that the child showed no preexisting preference for any character, the experimenter said: “Little Yellow, Little Red, and Little Blue are classmates. They all know what is inside the mystery boxes. Does that make you curious? Which of them would you like to ask about what is inside the mystery box?” The child’s choice—indicated by a pointing gesture or a verbal response—was recorded.

During the emotional-context phase, children were randomly assigned to either a happy or an unhappy scenario. The happy scenario referred to a positive emotional context in which the characters experienced a successful and enjoyable outcome, whereas the unhappy scenario referred to a negative emotional context in which the characters experienced a frustrating or disappointing outcome. For example, in the successful puzzle-solving happy scenario, the computer showed a short video of Little Yellow, Little Red, and Little Blue playing with a puzzle and finishing it. After completing the puzzle, the characters cheered and said, “We did it! We are so happy!” In the unsuccessful puzzle-solving unhappy scenario, the computer showed a short video in which Little Yellow, Little Red, and Little Blue tried to complete the puzzle but failed despite their efforts. The characters looked down, sighed, and said, “Oh no, we could not finish it. We are so sad.” To test children’s emotion recognition, the experimenter then asked, “Do you think they were happy or sad during their activity today?” The child’s response was recorded. All children included in the final analyses correctly identified the emotional valence of the scenario, indicating that the emotional-context manipulation was effective.

**Figure 1:**
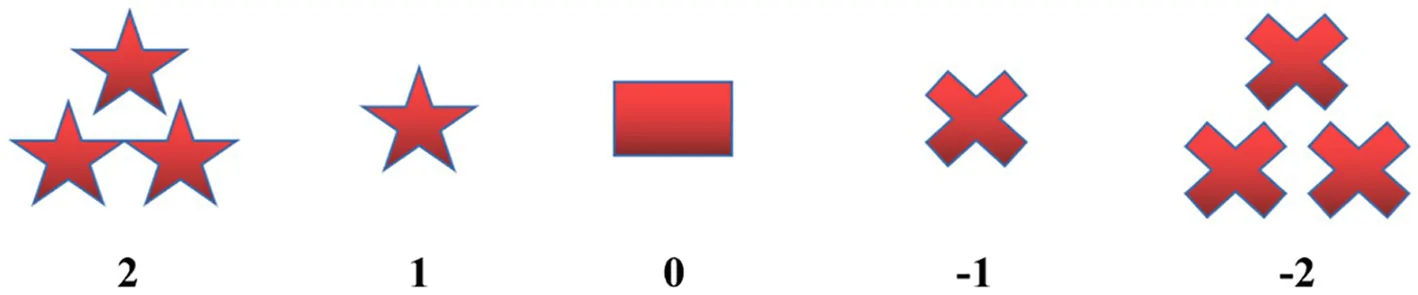
Moral evaluation tool. The patterns from left to right represent 2 points, 1 point, 0 points, −1 point, and −2 points, respectively.

During the gossip moral-rating phase—when the gossip target was absent—the spreader delivered positive or negative gossip to an audience at predetermined frequencies (66.7% positive, 66.7% negative, 100% positive, 100% negative). Transmission frequency was defined as the proportion of positive (or negative) testimonies delivered by the gossip spreader across the three statements within each scenario. We designed two frequency levels: 100% (all three statements were either all positive or all negative) and 66.7% (two out of three statements were positive or negative, with the remaining statement being neutral). The 66.7% level was chosen because, given a fixed total of three statements per scenario, it represents the natural majority level just below unanimity (100%). This manipulation aligns with Boseovski and Lee (2006), who showed that young children’s social judgments and behavioral predictions can be influenced by the amount of behavioral information available. The frequency manipulation was embedded in short video scenarios, and children naturally observed the sequence of testimonies rather than being explicitly told the frequency ratio. The display presented side-by-side images of Little Yellow and Little Blue, and the experimenter said: “One day, Little Yellow and Little Blue were talking about Little Red in the classroom while Little Red was swinging outside.” In a positive example, Little Yellow told Little Blue, “Did you hear Little Red found another child’s watch outside? She returned it immediately—she did the right thing.” The moral-evaluation instrument (Figure 1) was then displayed, and children were asked to rate the spreader’s behavior (e.g., “How would you rate Little Yellow’s behavior?”). In this phase, Little Yellow and Little Blue acted as spreaders across three distinct scenarios; each scenario required a separate moral rating.

**Figure 2:**
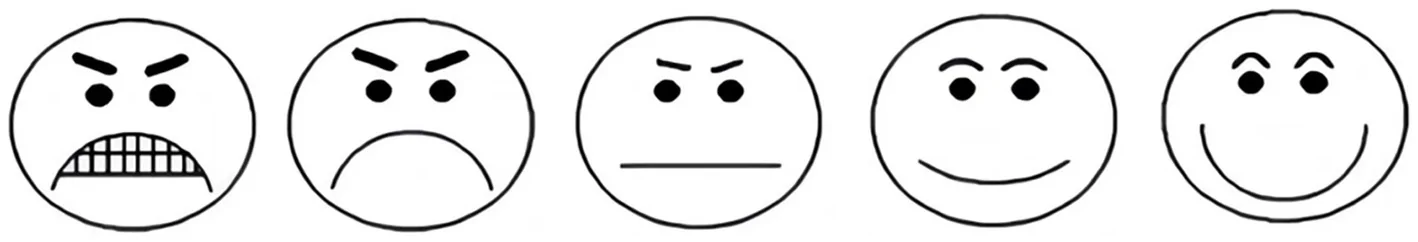
Attitude assessment tool. The patterns from left to right represent 1 point, 2 points, 3 points, 4 points, and 5 points, respectively.

During the evaluation phase, the attitude assessment instrument (Figure 2) was presented on the screen. Children were asked, in order, “Do you think Little Yellow/Little Blue/Little Red is a good kid?” They gave an attitude rating on a pictorial scale of frowning-to-smiling faces (1 = negative attitude to 5 = positive attitude), with higher scores indicating a more favorable attitude. Social preferences were then assessed using the questions “Who do you think is the friendliest and most trustworthy?” and “Which of them do you think has more friends?” To score, selections in the introduction and evaluation phases were coded as follows: choosing the positive gossip spreader = 2 points, choosing the target of the gossip = 1 point, and choosing the negative gossip spreader = 0 points. Scores from the two phases were summed, yielding a total social-preference score ranging from 0 to 4.

**Figure 3:**
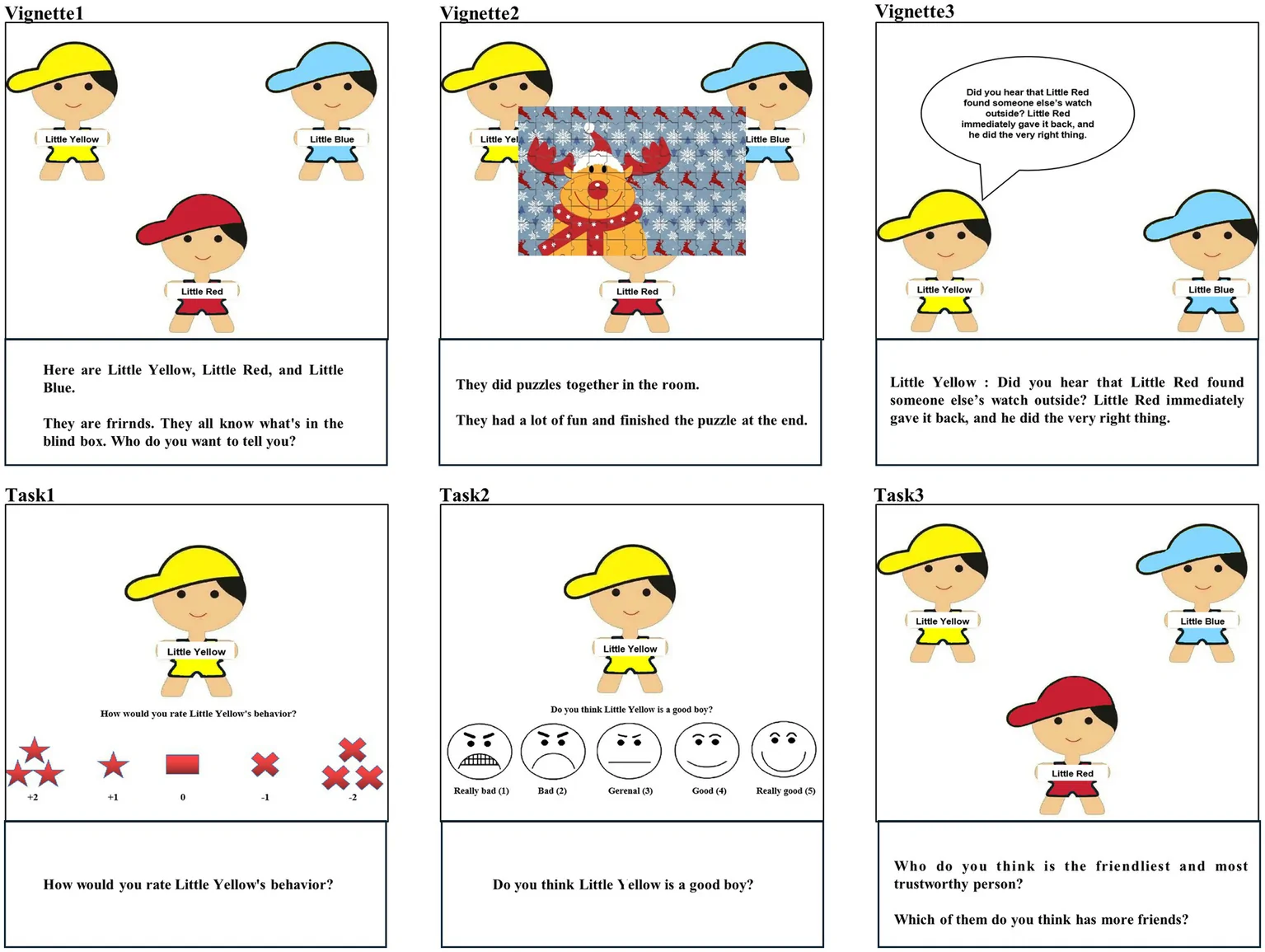
Specific flowchart for a single trial in Study 1. Different types of testimonies are tied to objects with fixed colors, namely the gossip spreader (Little Yellow—positive testimony), the subject of the gossip (Little Red—neutral testimony), and the gossip recipient (Little Blue—negative testimony). Adapted with permission from the illustration by Lanruier on Nipic, licensed under standard royalty-free license, Source: https://www.nipic.com/show/6111245.html.

We conducted one-on-one experiments with children aged 5–7 years. The specific flowchart for a single trial in Study 1 is shown in Figure 3.

## Results

To determine whether children displayed a pre-existing preference for the experimental characters during the introduction phase, we compared the number of times children chose those characters to the chance level (1 point) using one-sample Wilcoxon signed-rank tests. Results indicated no deviation from chance for the five-year-old (M = 0.80, SD = 0.73, *W* = 54, *p* = 0.225) and six-year-old groups (M = 0.90, SD = 0.67, *W* = 174, *p* = 0.450). In contrast, the seven-year-old group scored significantly below chance (M = 0.80, SD = 0.67, *W* = 72, *p* < 0.05), reflecting an initial bias against the experimental characters and a tendency to prefer blue prior to the main experiment.

### Children’s moral evaluations of gossip spreaders

Descriptive statistics and multiple linear regression analyses were performed to examine the moral judgments of children (ages 5–7) across different gossip−dissemination scenarios. Descriptive statistics are reported in Table 2, and regression results are presented in Table 3.

**Table 2:** Descriptive statistics results of moral evaluation and attitude evaluation in study 1.

Age	Frequency	Valence/scenario	M	SD
5	100%	Positive	4.63	1.22
		Negative	3.25	1.85
	66.7%	Positive	1.60	2.50
		Negative	1.60	2.39
6	100%	Positive	4.26	1.99
		Negative	0.06	3.79
	66.7%	Positive	2.45	1.71
		Negative	1.28	2.62
7	100%	Positive	4.50	1.41
		Negative	0.04	3.82
	66.7%	Positive	2.87	1.54
		Negative	1.87	2.91
5	100%	Happy	4.82	0.59
		Unhappy	4.20	0.79
	66.7%	Happy	3.80	1.70
		Unhappy	3.80	1.69
6	100%	Happy	4.77	0.43
		Unhappy	4.43	0.86
	66.7%	Happy	4.00	1.15
		Unhappy	3.83	1.23
7	100%	Happy	4.43	0.63
		Unhappy	4.90	0.31
	66.7%	Happy	4.31	0.84
		Unhappy	4.10	1.25

M, mean; SD, standard deviation.

**Table 3:** The results of the moral evaluation of the multiple linear regression model in Study 1.

Predictor	*b*	SE	*t*	*p*
Intercept	5.62	0.84	6.71	<0.001***
Age: 6–5	−1.06	0.95	−1.12	0.264
Age: 7–5	−0.96	1.00	−0.96	0.336
Gender	−0.97	0.85	−1.14	0.256
Emotional context	−0.22	0.91	−0.24	0.809
Frequency	−3.98	0.85	−4.69	<0.001***
Valence	−2.46	0.83	−2.95	<0.01**
Age: 6–5 × gender	0.74	0.87	0.85	0.395
Age: 7–5 × gender	0.79	0.90	0.88	0.381
Age: 6–5 × emotional context	−0.12	0.87	−0.14	0.888
Age: 7–5 × emotional context	−0.30	0.91	−0.33	0.742
Age: 6–5 × frequency	2.15	8.86	2.49	<0.05*
Age: 7–5 × frequency	2.65	0.89	2.97	<0.01**
Age: 6–5 × valence	−2.04	0.84	−2.44	<0.05*
Age: 7–5 × valence	−2.10	0.86	−2.44	<0.05*
Gender × emotional context	−0.05	0.65	−0.08	0.937
Gender × frequency	−0.37	0.64	−0.58	0.561
Gender × valence	0.69	0.63	1.10	0.274
Emotional context × frequency	0.43	0.65	0.67	0.505
Emotional context × valence	0.07	0.65	0.12	0.908
Frequency × valence	2.87	0.63	4.57	<0.001***

Significance: ***: <0.001; **: <0.01; *: <0.05.

To evaluate children’s evaluations of the moral quality of gossip-spreaders’ behavior, we fitted a multiple linear regression model that included all main effects and second-order (two-way) interaction terms. Gender, age, emotional context, gossip frequency, and gossip valence were entered as predictors, and moral−judgment scores served as the dependent variable.

Results indicate a significant main effect of gossip transmission frequency (*b* = −3.98, SE = 0.85, *t* = −4.69, *p*<0.001), with children assigning significantly higher moral evaluation scores to the gossip spreader’s behavior under high−frequency conditions compared to low-frequency conditions. The main effect of gossip valence was also significant (*b* = −2.46, *t* = −2.95, SE = 0.83, *p*<0.01), and children’s moral evaluation scores for gossip spreaders’ behavior were significantly higher under positive testimony conditions than under negative testimony conditions.

The interaction between children’s age (age 6 vs. 5: *b* = 2.15, SE = 0.86, *t* = 2.49, *p* < 0.05; age 7 vs. 5: *b* = 2.65, SE = 0.89, *t* = 2.97, *p* < 0.01) and gossip frequency was significant. *Post-hoc* analysis revealed that 5-year-olds scored significantly higher on moral judgments under high-frequency conditions than low-frequency ones [*d* = −2.34, 95% CI (−4.49, −0.19), *p* < 0.05], while no significant differences were observed among 6-year-olds [*d* = −0.30, 95% CI (−1.81, 1.21), *p* = 0.993] and 7-year-olds [*d* = −0.10, 95% CI (−1.65, 1.84), *p* = 1.000].

The interaction between children’s age (age 6 vs. 5: *b* = −2.04, SE = 0.84, *t* = −2.44, *p* < 0.05; age 7–5: *b* = −2.10, SE = 0.86, *t* = −2.44, *p* < 0.05) and gossip valence was significant. Post-hoc analyses revealed that children aged 6 [*d* = −2.81, 95% CI (−4.20, −1.42), *p* < 0.001] and 7 [*d* = −2.77, 95% CI (−4.38, −1.15), *p* < 0.001] scored higher on moral judgments under the positive testimony condition than under the negative testimony condition, whereas no significant difference was observed among 5-year-olds [*d* = −0.71, 95% CI (−2.70, 1.28), *p* = 0.909].

Additionally, the interaction between gossip frequency and valence was significant (*b* = 2.87, SE = 0.63, *t* = 4.57, *p* < 0.001). Under high-frequency transmission conditions, children’s moral ratings for positive testimonies were significantly higher than for negative testimonies [*d* = −3.68, 95% CI (−4.80, −2.55), *p* < 0.001], whereas no significant difference was observed under low−frequency conditions [*d* = −0.85, 95% CI (−2.04, 0.34), *p* = 0.251].

**Figure 4:**
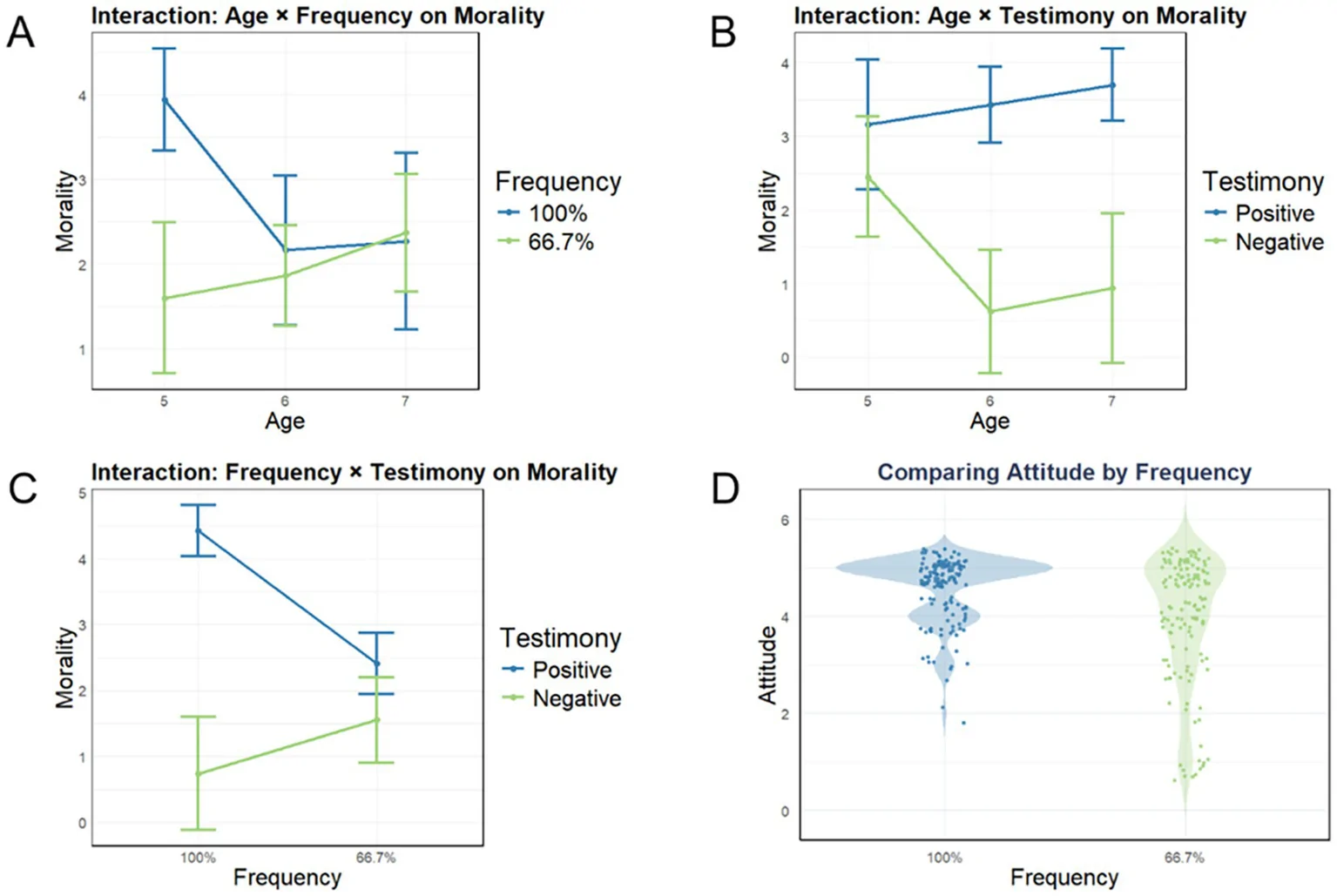
**(A)** Interaction between age and frequency on moral judgment scores. **(B)** Interaction between age and testimony valence on moral judgment scores. **(C)** Interaction between frequency and testimony valence on moral judgment scores. **(D)** Comparison of attitudes toward the target between high-frequency (100%) and low-frequency (66.7%) conditions.

All other main effects and interactions were insignificant. Interaction results are illustrated in Figure 4.

### Children’s attitude evaluations toward gossip spreaders

Descriptive statistics and multiple linear regression analyses were conducted on data concerning the attitudes of children (ages 5–7) toward gossip spreaders. Descriptive statistics are reported in Table 2.

To examine children’s cognitive attitudes toward gossip spreaders, we fitted a multiple linear regression model that included all main effects and second-order (two-way) interaction terms. Gender, age, situational state, gossip frequency, and valence were entered as predictors, and attitude scores served as the dependent variable.

Results showed a significant main effect of gossip frequency (*b* = −0.95, SE = 0.30, *t* = −3.21, *p* < 0.01), such that children’s attitude scores toward gossip spreaders were lower in the low−frequency condition than in the high-frequency condition.

All other main effects and interactions were insignificant.

### Children’s social preferences

Comparing the total scores of children on two items during the evaluation phase with the random level (2 points), the results of the one-sample Wilcoxon signed-rank test revealed no significant differences in scores for friendly and social preferences (5-year-old group: M = 2.40, SD = 1.18, *W* = 154.00, *p* = 0.059). However, significant differences were observed in scores for friendly and social preferences among 6-and 7-year-old children (6-year-old group: M = 3.00, SD = 1.05, *W* = 1119.00, *p* < 0.001; 7-year-old group: M = 2.70, SD = 1.20, *W* = 455.50, *p* < 0.001) with scores significantly higher than random levels. This indicates that children aged 5–7 years exhibit greater expectations and willingness to befriend positive gossip spreaders.

## Discussion

In Experiment 1, we examined factors influencing moral evaluations, attitudinal judgments, and social preferences toward gossip spreaders among 5-to 7-year-old children. The results revealed age-related changes in how children process information about gossip transmission, with these processes further moderated by situational factors—specifically transmission frequency and the content of valence.

First, 5-year-olds displayed a qualitatively different pattern of moral judgment compared with older children: their evaluations were driven more strongly by transmission frequency, such that frequent gossip spreaders received higher moral ratings. By contrast, 6-to 7-year-olds gave significantly higher moral ratings to actors who conveyed positive testimony, increasingly integrating motivational and valence cues into their judgments. This developmental shift corresponds with the maturation of theory of mind: older children can infer communicative intent and form more nuanced moral judgments incorporating actors’ intentions and motivations, whereas younger children rely more on salient behavioral cues like frequency (Buon and Margoni, 2025).

When frequency and valence interact, children’s moral judgments become more nuanced. For high-frequency gossip spreaders, gossip valence drives moral evaluations: children give higher moral ratings when frequent spreading is accompanied by positive testimonials, but they judge equally frequent gossiping more harshly when testimonials are negative. These findings support Hypothesis 1, indicating that children assign higher moral evaluations to individuals who spread positive gossip and are more willing to befriend them. One possible explanation is that children view repeated spreading as a signal of strong commitment; its moral status therefore depends less on frequency alone than on whether the spreader’s motives are framed positively (Carlson et al., 2022). Frequency also affected children’s attitudinal evaluations: participants rated 100% frequency gossip spreaders more positively than those who gossiped at 66.7% frequency. This effect may reflect increased credence from repetition, such that higher-frequency spreaders were judged more trustworthy and thus received higher attitude scores (Udry and Barber, 2024).

In the initial preference phase of Experiment 1, only the 7-year-olds showed a color preference for blue, whereas the 5- and 6-year-olds did not. If color associations had driven moral evaluations, all age groups would have shown a consistent pattern. Instead, only the 7-year-olds exhibited a color preference, whereas both 6- and 7-year-olds distinguished between positive and negative spreaders based on valence. This suggests that color preferences alone cannot account for the age × valence interaction observed in this study. Overall, the results of Experiment 1 suggest that with age, children gradually shift from relying on surface cues to integrating motivational and valence information when evaluating gossip spreaders, a developmental transition closely related to the maturation of theory of mind.

### Study 2

Study 1 examined young children’s attitudes toward gossip spreaders, moral judgments, and friendship intentions across age groups and gossip contexts. Study 2 employed a trust-game paradigm to assess children’s trust and evaluative judgments of gossip spreaders and recipients, manipulating reputation and relationship under negative gossip conditions.

## Method

### Participants

Using G*Power 3.1.9.7 with power set at 0.80 and a medium effect size (*f* = 0.25), the minimum required sample was estimated as *N* = 89. Based on the age effects observed in Study 1, we restricted the age range of Study 2 to 5- and 6-year-olds in order to focus on the critical developmental period. Specifically, a significant developmental transition occurred between age 5 and age 6 in the moral evaluation task: 6-year-olds were able to distinguish gossip spreaders by valence, whereas 5-year-olds were not. No comparable qualitative shift was observed between age 6 and age 7. Accordingly, we recruited 108 children (ages 5–6) from a kindergarten. None of the participants had taken part in Study 1, and all completed Study 2; therefore, no data were excluded. The final sample consisted of 108 participants (M_age_ = 5.53 years, SD = 0.50), with descriptive statistics reported in Table 4 and group assignments reported in Supplementary Table S3. Written parental consent and verbal child assent were obtained before testing.

**Table 4:** Demographic information of the Study 2 sample by age group.

Age group	*N*	Mean age	SD	Male	Female
5-year-olds	51	5.00	0.00	31	20
6-year-olds	57	6.00	0.00	33	24
Total	108	5.53	0.50	64	44

*N*, number of participants; SD, standard deviation.

### Procedure

The procedure was adapted from the Trust Game paradigm (Berg et al., 1995). Prior to testing, experimenters explained the procedure to teachers and children, and told children they would receive a gift after completing the trials. Study 2 used the same negative life events and stimuli as Study 1; each participant completed 6 trials. Each trial comprised five stages: practice, introduction, questioning, social-context, and evaluation. The first three stages replicated those in Study 1. The key constructs in Study 2 were trust behavior, attitude evaluation, relationship type, reputation, and target role. Definitions, operationalizations, example manipulations, and scoring procedures for these constructs are summarized in Supplementary Table S2.

During the social-context stage, the relationships among the experimental characters and the reputation of the gossip spreader were introduced. We confirmed their understanding of the relationship by explaining to the children that “Little Yellow, Little Blue, and Little Red are classmates who often share food and study together” or “Little Yellow, Little Blue, and Little Red are strangers meeting for the first time today.” Across the six trials, Little Blue consistently provided Little Yellow with negative testimony about Little Red. To assess belief they were asked, “Do you believe what Little Blue said?” After recording this response, children were informed, “A school investigation found that Little Blue’s information was true/false. Little Red did/did not do that thing.” The belief question was then repeated.

The evaluation stage comprised measurement of trust and of attitudes. Trust was assessed using 12 rounds of a trust game. Each round began with a 2-s fixation cross, followed by a 5-s presentation of a image (Little Blue or Little Red) and the instruction that the person on screen was the partner for that round. After a 2-s consideration period, the child decided how many coins (0–10) to give the partner; the number of coins reported verbally by the child (and entered by the experimenter) served as the trust score for that partner (0 = low trust, 10 = high trust). Following each round, the computer screen displayed the feedback results, informing the participant in an understandable manner: “The other participant reported you have ×× yuan. You currently have ×× yuan.” Finally, children completed an attitude−evaluation task in which they rated the target’s overall behavior in the preceding scenario on a 1–5 pictorial scale (Figure 2). The scale ranged from a frowning face (1 = poor attitude) to a smiling face (5 = excellent attitude); higher scores indicated more positive attitudes.

**Figure 5:**
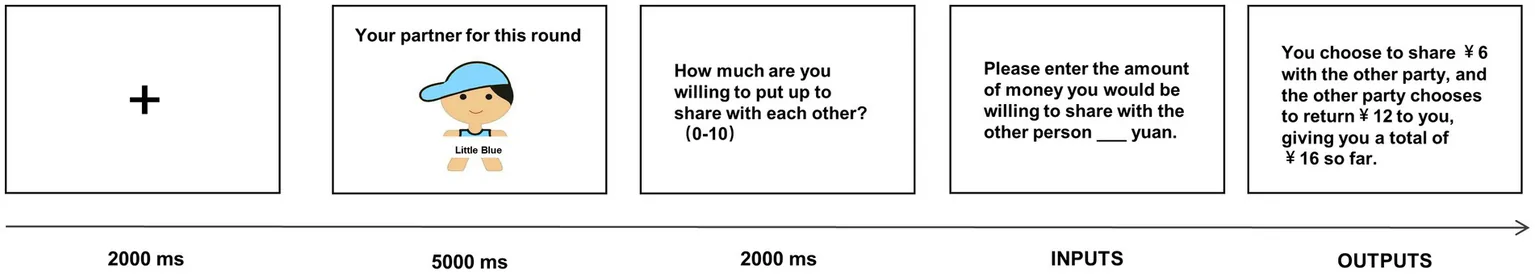
Specific flowchart for single−trial trust data collection in Study 2. Adapted with permission from the illustration by Lanruier on Nipic, licensed under standard royalty-free license, Source: https://www.nipic.com/show/6111245.html.

The specific flowchart for collecting single−trial trust data in Study 2 is shown in Figure 5.

## Results

To determine whether children showed baseline preferences for the experimental characters, we compared the number of times children aged 5–6 selected each character during the introduction phase to the chance level (1 point) using one−sample Wilcoxon signed−rank tests. These tests revealed no significant deviation from chance for either age group (5-year-olds: M = 1.17, SD = 0.78, *W* = 330.00, *p* = 0.16; 6-year-olds: M = 1.10, SD = 0.83, *W* = 420.00, *p* = 0.63).

### Young children’s trust in gossip spreaders

Descriptive statistics and linear mixed-effects models were used to examine the trust levels of 5- to 6-year-old children toward different roles in gossip propagation across gossip contexts. We fitted a mixed-effects model that included all main effects and second order interactions, with gender, age, social relationship, reputation, and target character as fixed effects and trust scores as the dependent variable. Descriptive statistics appear in Table 5, and the linear mixed-model results are reported in Table 6.

**Table 5:** Descriptive statistics for trust scores in Study 2 (M ± SD).

Age	Relationship	Reputation	Sender	Target	Receiver
5	Friend	High	6.02 ± 3.51	6.45 ± 3.41	6.54 ± 3.55
		Low	4.23 ± 2.16	5.00 ± 2.90	4.95 ± 2.47
	Strangers	High	5.05 ± 3.28	3.70 ± 3.30	5.18 ± 3.10
		Low	4.85 ± 3.60	5.62 ± 3.46	5.70 ± 3.41
6	Friend	High	6.09 ± 2.97	4.83 ± 3.20	5.43 ± 3.26
		Low	4.80 ± 3.01	5.37 ± 2.92	4.61 ± 3.03
	Strangers	High	4.45 ± 2.27	3.03 ± 2.83	4.63 ± 2.69
		Low	2.24 ± 2.41	3.74 ± 3.03	3.10 ± 2.52

M, mean; SD, standard deviation.

**Table 6:** Results of the linear mixed model for trust scores in Study 2.

Fixed effects	*b*	SE	*t*	*p*
(Intercept)	6.35	0.73	8.66	<0.001***
Target character: target-sender	−0.64	0.38	5.48	0.089
Target character: receiver–sender	0.16	0.38	0.44	0.662
Gender	−0.33	0.89	−0.37	0.709
Relationship	−0.82	0.87	−0.94	0.347
Reputation	−1.53	0.88	−1.74	0.084
Age	−0.18	0.86	−0.21	0.834
Target character: target-sender × gender	0.09	0.34	0.28	0.781
Target character: receiver-sender × gender	−0.19	0.34	−0.56	0.579
Target character: target-sender × relationship	−0.04	0.34	−0.11	0.916
Target character: receiver–sender × relationship	0.38	0.34	1.13	0.260
Target character: target-sender × reputation	1.65	0.34	4.89	<0.001***
Target character: receiver–sender × reputation	0.58	0.34	1.71	0.088
Target character: target-sender × age	−0.33	0.34	−0.97	0.330
Target character: receiver–sender × age	−0.48	0.34	−1.44	0.151
Gender × relationship	−0.42	0.88	−0.48	0.635
Gender × reputation	−0.36	0.89	−0.41	0.686
Gender × age	0.58	0.88	0.65	0.515
Relationship × reputation	0.62	0.86	0.72	0.472
Relationship × age	−1.17	0.85	−1.37	0.174
Reputation × age	0.10	0.87	0.12	0.906

Significance: ***: <0.001; **: <0.01; *: <0.05.

The main effect of target character was significant, with gossip target scores significantly lower than gossip-sender (*b* = −0.64, SE = 0.38, *t* = 5.48, *p* = 0.089); the difference between gossip-receiver and gossip-sender was not significant (*b* = 0.16, SE = 0.38, *t* = 0.44, *p* = 0.662). Additionally, the main effects of gender, social relationships, reputation, and age were all insignificant.

**Figure 6:**
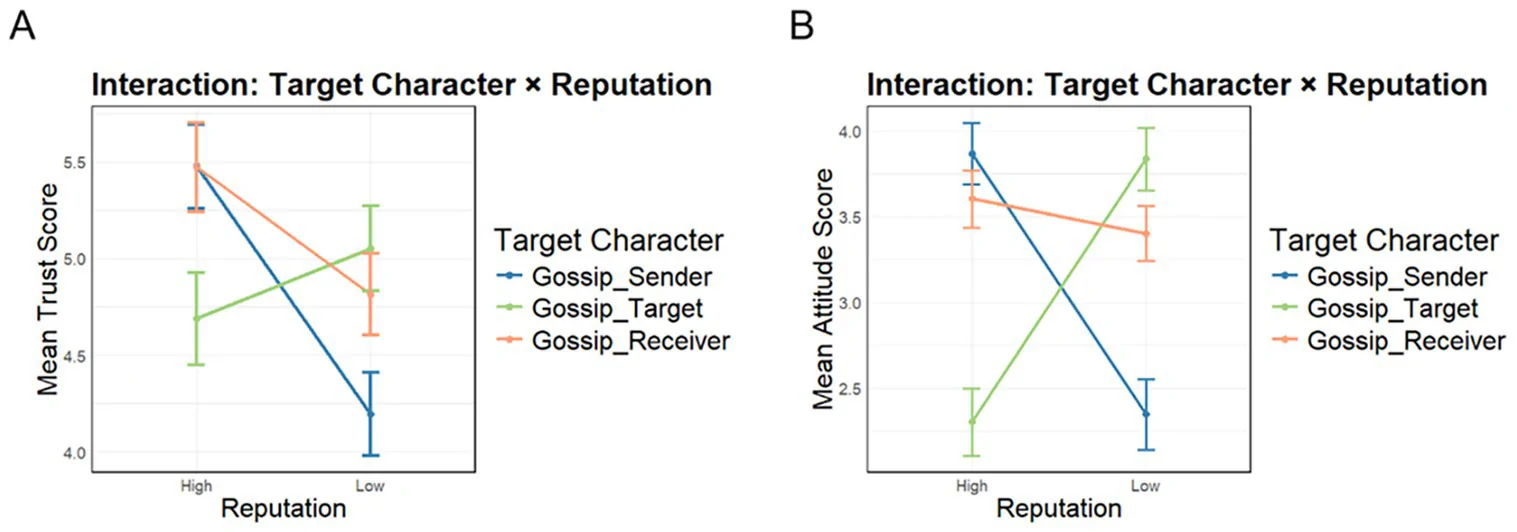
**(A)** Interaction between the target character and reputation on trust scores. **(B)** Interaction between the target character and reputation on attitude scores.

Target character (target-sender: *b* = 1.65, SE = 0.34, *t* = 4.89, *p* < 0.001; receiver-sender: *b* = 0.58, SE = 0.34, *t* = 1.71, *p* = 0.088) showed a significant interaction with reputation (Figure 6). Specifically, in the high-reputation condition, gossip-sender scores were significantly higher than gossip−target (*b* = 0.78, SE = 0.24, *t* = 3.22, *p* < 0.05), while gossip-target scores were significantly lower than gossip−receiver (*b* = −0.79, SE = 0.24, *t* = −3.29, *p* < 0.05). The difference in scores between gossip-sender and gossip-receiver was not significant (*b* = −0.02, SE = 0.24, *t* = −0.07, *p* = 0.997).

In low-reputation scenarios, gossip-sender scores were significantly lower than gossip-target (*b* = −0.88, SE = 0.24, *t* = −3.65, *p* < 0.001) and significantly lower than gossip-receiver (*b* = −0.60, SE = 0.24, *t* = −2.48, *p* < 0.05). The difference in scores between gossip-target and gossip-receiver was not significant (*b* = 0.28, SE = 0.24, *t* = 1.18, *p* = 0.468).

### Young children’s attitude evaluation of experimental subjects

Descriptive statistics and linear mixed-effects model analyses were conducted to examine how 5-to-6-year-old children evaluated different roles in gossip propagation across gossip contexts.

We first fitted a linear mixed-effects model that included all main effects and second-order interaction terms, with gender, age, social relationship, reputation, and target of evaluation as fixed effects and attitude-evaluation scores as the dependent variable. Descriptive statistics are reported in Table 7, and the linear mixed-effects model results are presented in Supplementary Table S4.

**Table 7:** Descriptive statistics results of attitude evaluation in Study 2 (M ± SD).

Age	Relationship	Reputation	Sender	Target	Receiver
5	Friend	High	3.50 ± 1.51	2.79 ± 1.67	3.86 ± 1.35
		Low	2.18 ± 1.40	3.36 ± 1.63	3.45 ± 0.82
	Strangers	High	4.00 ± 1.18	2.00 ± 1.18	3.73 ± 1.19
		Low	2.73 ± 1.53	3.87 ± 1.06	3.33 ± 1.18
6	Friend	High	4.40 ± 1.12	2.40 ± 1.64	3.27 ± 1.22
		Low	2.33 ± 1.84	4.33 ± 1.40	3.60 ± 1.35
	Strangers	High	3.54 ± 1.27	1.92 ± 1.04	3.62 ± 1.12
		Low	2.07 ± 1.38	3.64 ± 1.28	3.21 ± 1.31

M, mean; SD, standard deviation.

The main effect of reputation was significant (*b* = 1.83, SE = 0.37, *t* = −4.90, *p*<0.001), with children scoring higher in the high-reputation condition than in the low-reputation condition. Furthermore, the main effects of gender, social relationships, and age were not significant.

The interaction between target character (target-sender: *b* = 3.07, SE = 0.37, *t* = 8.19, *p* < 0.001; receiver-sender: *b* = 1.29, SE = 0.37, *t* = 3.46, *p* < 0.001) and reputation was significant (Figure 6). Specifically, in the high-reputation condition, the gossip-target score was significantly lower than that of the gossip-sender (*b* = 1.55, SE = 0.27, *t* = 5.77, *p* < 0.001) and also significantly lower than that of the gossip-receiver (*b* = −1.30, SE = 0.27, *t* = −4.81, *p* < 0.001). The difference in scores between gossip-sender and gossip-receiver was not significant (*b* = 0.26, *SE* = 0.27, *t* = 0.95, *p* = 0.608).

In low-reputation scenarios, the gossip-target scored significantly higher than the gossip-sender (*b* = −1.51, SE = 0.27, *t* = −5.72, *p* < 0.001) and significantly higher than the gossip-receiver (*b* = −1.04, SE = 0.27, *t* = −3.92, *p* < 0.001). The difference in scores between gossip-target and gossip-receiver was not significant (*b* = 0.48, SE = 0.27, *t* = 1.80, *p* = 0.173).

## Discussion

In Experiment 2, we investigated the factors of trust and attitude evaluations among 5 -to 6-year-old children across different roles within negative gossip dissemination scenarios. The results supported Hypothesis 2, showing that children were less likely to trust gossip spreaders with a negative reputation. However, children’s relationship with the gossip spreader (i.e., peer vs. stranger) did not significantly influence their judgments. One possible explanation is that the experimental setting did not involve real interactions with actual peers or strangers, which may account for the lack of support for Hypothesis 3.

Notably, under negative gossip conditions, trust scores and attitude evaluations demonstrated remarkable consistency in their patterns across roles: reputation reversals elicited corresponding reversals in both trust and attitude assessments. A particularly striking pattern emerged in the high-reputation condition, wherein the gossip sender garnered the highest trust and attitude ratings, followed by the gossip receiver, with the gossip target receiving the lowest evaluations. This pattern was reversed in the low-reputation condition. When endowed with a strong reputation, the sender commanded greater trust from children (sender > receiver > target), a pattern that aligns with theoretical perspectives positioning high-reputation individuals as reliable information sources. Conversely, when the sender’s reputation was compromised, children’s trust in the sender declined substantially and, in some cases, transferred to the target. This reallocation of trust demonstrates children’s sophisticated understanding of how reputation functions as a critical regulator of social interactions within gossip contexts.

These findings provide compelling empirical support for Social Information Processing Theory, which posits that children learn social rules and expectations by observing others’ behavior and its consequences. Within this theoretical framework, high-reputation communicators are presumed to furnish accurate, valuable information, whereas low reputation is associated with unreliability or dishonesty, reducing trust (Clément, 2010; Turchio and Evans, 2024).

Finally, the correlation between trust and attitude scores reveals that children’s trust allocations within these social contexts are intrinsically connected to their evaluative judgments. This finding of behavioral and attitudinal responses supports the robustness of the model: reputation not only affects direct trust-related behavior but also shapes evaluative judgments by observers.

### General discussion

Study 1 systematically examined children’s attitudes, moral judgments, and friendship preferences toward gossip spreaders across multiple age groups and gossip contexts. Study 2 narrowed the focus to negative gossip scenarios while expanding outcome measures to include children’s trust and attitude evaluations toward gossip senders, recipients, and targets, who varied in reputation and social relationship, as assessed through a trust-game paradigm.

Across both experiments, 5- and 6-year-olds showed distinct patterns of evaluation for gossip-related behavior, contingent on specific task demands. In moral judgment tasks (Experiment 1), 6-year-olds integrated communicative motives, assigning higher moral value to frequent gossip when driven by prosocial intentions. In contrast, 5-year-olds, based moral judgments primarily on transmission frequency. In the multi-role trust-evaluation task (Experiment 2), however, both age groups behaved similarly: children redistributed trust according to the reputations of the different roles. These findings indicate that children’s understanding of gossip dynamics does not follow a single, linear developmental trajectory but emerges from the interplay of multiple cognitive processes engaged by contextual demands.

The results from Experiment 1 suggests that 5-year-olds have limited capacity to integrate complex motives when forming moral judgments about gossip, reflecting constraints in higher-order cognitive interpretation. Paradoxically, in Experiment 2, the same 5-year-olds adjusted trust allocations as precisely as 6-year-olds in trust-based decisions. This dissociation suggests that intuitive social risk-assessment mechanisms, which supports reputationally guided trust behavior, may develop earlier than the cognitive capacities required for motive-based moral reasoning.

This task-dependent dissociation can be better understood through the lens of the dual systems model in developmental cognitive neuroscience. According to this model, individual behavior is driven by two interacting systems: an earlier-maturing “socioemotional-incentive processing system,” which is highly sensitive to novelty, reward, and arousing stimuli, and a later-developing, linearly maturing “cognitive control system,” which supports inhibitory control and the regulation of impulsive behavior. The developmental asynchrony between these two systems explains an important developmental phenomenon: even when logical reasoning and information-processing abilities approach adult levels by early adolescence, actual decision-making in high-risk contexts often remains markedly impulsive. This occurs because the earlier-maturing reward system dominates at this stage, while the still-developing cognitive control system is not yet sufficiently mature to consistently constrain its influence (Shulman et al., 2016).

Extending this framework to earlier developmental stages, the socioemotional system’s functional maturity may emerge long before the cognitive control system reaches full capacity. Indeed, evidence from infancy suggests that social evaluation abilities are present very early in life. Supporting this, Hamlin et al. (2007) found that 12-month-old infants, though pre-linguistic, already show selective social preferences: they approach “helpers” who act prosocial and avoid “obstructors” who behave antisocially. Such early differentiation facilitates alliance formation with trustworthy partners and avoidance of social threats, promoting adaptive developmental outcomes. In the context of the present study, this early-emerging socioemotional system may underpin 5-year-olds’ ability to adjust trust based on reputation cues in Experiment 2, whereas the cognitive control demands of integrating motives in moral evaluation (Experiment 1) may still be developing at this age.

The age-related differences in moral judgment observed in Experiment 1 may map onto the development of theory of mind. Ochoa et al. (2023) demonstrated that the development of moral judgment is closely related to maturation of theory-of-mind abilities, which enables children to represent others’ beliefs and desires and integrate intentions with outcomes in moral evaluations. Although children as young as four to 5 years understand that others hold different mental states, the capacity to infer intentions in complex social contexts and to apply those in moral reasoning continues to refine into middle childhood. This prolonged development corresponds with maturation in brain regions involved in social cognition, particularly the dorsomedial prefrontal cortex (DMPFC) (Bowman et al., 2019).

Together, the findings of both experiments suggest that children’s responses to gossip are shaped by the interaction of evolutionarily early social-evaluation tendencies and later-developing cognitive capacities. Even with an incomplete conceptual understanding of gossip, children can make adaptive decisions in social situations—strategically seeking benefits and avoiding harm—by relying on intuitive assessments of social risk and reward.

### Limitations

Although this study extends prior work and advances our understanding of children’s gossip behavior, several limitations should be acknowledged. First, we manipulated only emotional valence (positive vs. negative) and did not examine the effects of discrete emotions. Future research should probe how specific emotions (e.g., surprise, disgust, anger, sadness) differentially influence children’s evaluations of gossip.

Second, the ecological validity of our experimental paradigm is limited. Subsequent studies should employ more naturalistic social-communication scenarios (e.g., interactive role-plays, peer-group settings, or longitudinal observations) to assess how contextual cues interact with gossip content.

Third, the cross-sectional design precludes inferences about individual developmental trajectories. Longitudinal or cross-sequential designs are needed to identify critical turning points and factors that shape the emergence and refinement of gossip-recognition and evaluation abilities.

Fourth, the assignment of character colors was not counterbalanced with the valence of the testimony. Future studies should counterbalance the pairing of colors with testimony valence to rule out any potential implicit color biases.

Fifth, because Experiments 1 and 2 employed different paradigms and different sets of variables, the present study cannot directly compare the temporal relationship between children’s behavioral decisions and their cognitive evaluations. Behavioral decisions refer to children’s actual investment choices in the Trust Game in Experiment 2, namely the number of coins they gave to the trustee. Cognitive evaluations refer to children’s explicit moral ratings and attitude ratings in both experiments. Future research should examine both trust behavior and evaluative judgments within the same task paradigm to determine how these two processes relate to each other developmentally.

### Implications for research and practice

This study moves beyond a simple focus on gossip valence to show how gossip effects are moderated by contextual factors, notably the communicator’s reputation and the social relationship among actors. These findings highlight the importance of integrating multiple—and sometimes conflicting—social cues in future research to clarify how children weigh and synthesize complex social information in naturalistic interactions.

The results also carry practical implications for parents and educators concerned with children’s social–emotional development. Our study reveals that even five-year-olds can make nuanced trust decisions based on reputation, suggesting that outright prohibition of gossip may be less effective than guided discussion. Caregivers and teachers can use gossip-related incidents as teachable moments to discuss empathy, communicative motives, and the social consequences of sharing information, thereby supporting the development of theory of mind and moral reasoning.

In an era where children encounter online gossip early, our results underscore the need for early educational interventions. Such programs should encourage children to consider the source of information, its reputation, and the relevant social context; to analyze speakers’ motives; and to balance trust with critical evaluation when facing negative information about peers. Finally, given children’s sensitivity to reputation, educators can harness this tendency by cultivating classroom norms that reward prosocial behavior and thereby reduce the incidence and impact of harmful gossip.

## Conclusion

This study examines how children aged 5–7 evaluate different roles and make trust decisions in various gossip scenarios, revealing the Cognitive Development Process of children’s understanding of the gossip ecosystem. This process is shaped by the combined influence of evolutionary instincts and acquired cognitive development. Specifically: First, age 6 marks a critical turning point in moral judgment, after which children can integrate communicator intent for complex evaluations. Second, even at age 5, children can make Trust Decision-Making based on reputation cues. Third, younger children exhibit “behavioral decision-making preceding cognitive evaluation,” highlighting the primacy of intuitive systems in early social adaptation.

## Data Availability

The raw data supporting the conclusions of this article will be made available by the authors, without undue reservation.
