# Correction: Trust tangles: Who do kids trust? Unraveling young children's attitudes in the gossip mill

**DOI:** 10.3389/fpsyg.2026.1972285

**Published:** 2026-09-01

**Authors:** Songsong Wang, Xinru Wang, Weixian Liang, Lei Dai, Jinyan Song, Xiaobo Gao, Junting Huang, Xinyi Yu, Hongmin Bao, Qiang Zhou, She Zheng

**Affiliations:** 1 Department of Psychology, Wenzhou Medical University, Wenzhou, China; 2 School of Foreign Language Studies, Wenzhou Medical University, Wenzhou, China

**Keywords:** gossip, interpersonal trust, moral evaluation, social preference, young children

The funder College Students' Innovative Entrepreneurial Training Plan Program, S202510343061 was erroneously omitted.

The original version of this article has been updated.

