# Supplementary material for: Trust tangles: Who do kids trust? Unraveling young children’s attitudes in the gossip mill
Source: Front Psychol. 2026 Jul 22;17:1852349. doi: 10.3389/fpsyg.2026.1852349 (PMC13439614; doi:10.3389/fpsyg.2026.1852349)
Supplement: Supplementary file 1 [file Supplementary_file_1.DOCX]

**Table S1**

*Grouping of participants in Study 1*

| **Scenario** | **Frequency** | ***N*** | ***M±SD* (months)** | **Male** | **Female** |
| --- | --- | --- | --- | --- | --- |
| Happy | 100% positive/negative testimony | 38 | 72.96 ± 9.84 | 17 | 21 |
|  | 66.7% positive/negative testimony | 34 | 73.08 ± 9.96 | 19 | 15 |
| Unhappy | 100% positive/negative testimony | 36 | 73.68 ± 7.68 | 18 | 18 |
|  | 66.7% positive/negative testimony | 33 | 73.80 ± 8.76 | 19 | 14 |

*Note.* *N* number of participants, *M* mean, *SD* standard deviation.

**Table S2**

*Summary of All Constructs, Manipulations, and Scales for Study 1 and Study 2*

| Study | Construct | Definition | How Measured/Manipulated | Scale |
| --- | --- | --- | --- | --- |
| Study1 | Moral evaluation | Judgment of the gossip spreader's behaviour | Child rates "How would you rate Little Yellow's behaviour?" after hearing gossip | Pictorial scale from -2 (three crosses, very bad) to +2 (three stars, very good) |
| Study1 | Attitude evaluation | General attitude toward the gossip spreader as a person | Child answers "Do you think Little Yellow is a good kid?" | 1 (frowning face, very negative) to 5 (smiling face, very positive) |
| Study1 | Social preference (friendship) | Willingness to befriend the spreader and perception of spreader's popularity | Two questions: "Who is the friendliest and most trustworthy?" and "Who has more friends?" | Scoring: positive spreader = 2, target = 1, negative spreader = 0; sum = 0-4 |
| Study1 | Emotional context | Happy vs. unhappy scenario in which gossip occurs | Children randomly assigned to happy or unhappy context | Between-subjects manipulation |
| Study1 | Transmission frequency | How often the spreader conveys positive vs. negative testimony | 100% (all three statements same valence) vs. 66.7% (two same, one opposite) | Between-subjects manipulation |
| Study1 | Gossip valence | Positive vs. negative testimony about the target | Positive: "Little Red returned a lost watch"; Negative: "Little Red kicked a trash can" | Within-subjects manipulation |
| Study2 | Trust score | Amount of coins the child invests in the trustee (gossip spreader or target) in the trust game | Child decides how many of 10 coins to give to the partner | 0 (low trust) - 10 (high trust) |
| Study2 | Attitude evaluation | General attitude toward the gossip spreader or target after the scenario | Child rates "How would you rate Little Blue's / Little Red's overall behaviour?" | 1 (frowning face) - 5 (smiling face) |
| Study2 | Reputation | Spreader's honesty based on school investigation | High reputation: "Investigation found the gossip was true"; Low reputation: "Investigation found the gossip was false" | Between-subjects manipulation |
| Study2 | Social relationship | Whether the gossip spreader and target are friends or strangers | Friend condition: "They are classmates who often share food and study together"; Stranger condition: "They are meeting for the first time" | Between-subjects manipulation |
| Study2 | Target character | Role of the partner in the trust game | The partner shown on screen is either Little Blue (spreader) or Little Red (target) | Within-subjects manipulation |

**Table S3**

*Grouping of participants in Study 2*

| **Relationship** | **Reputation** | ***N*** | ***M*_age_±*SD* (months)** | **Male** | **Female** |
| --- | --- | --- | --- | --- | --- |
| Friend | High | 29 | 62.03 ±7.49 | 16 | 13 |
|  | Low | 26 | 60.65 ± 6.40 | 14 | 12 |
| Strangers | High | 24 | 62.08 ± 7.24 | 13 | 11 |
|  | Low | 29 | 60.59 ± 6.08 | 21 | 8 |

*Note.* *N* number of participants, *M* mean, *SD* standard deviation.

**Table S4**

*Results of the linear mixed model for attitude evaluation in Study 2*

| **Fixed Effects** | ***b*** | ***SE*** | ***t*** | ***p*** |
| --- | --- | --- | --- | --- |
| (Intercept) | 4.00 | 0.35 | 11.55 | ＜.001*** |
| Target character: Target-Sender | −1.45 | 0.42 | −3.50 | ＜.001*** |
| Target character: Receiver-Sender | −0.06 | 0.42 | −0.15 | 0.881 |
| Gender | −0.32 | 0.38 | −0.85 | 0.398 |
| Relationship | 0.17 | 0.38 | 0.44 | 0.659 |
| Age | 0.01 | 0.37 | 0.03 | 0.977 |
| Reputation | −1.83 | 0.37 | −4.90 | ＜.001*** |
| Target character: Target-Sender×Gender | 0.10 | 0.38 | 0.27 | 0.785 |
| Target character: Receiver-Sender×Gender | −0.17 | 0.38 | −0.44 | 0.660 |
| Target character: Target-Sender×Reputation | 3.07 | 0.37 | 8.19 | ＜.001*** |
| Target character: Receiver-Sender×Reputation | 1.29 | 0.37 | 3.46 | ＜.001*** |
| Target character: Target-Sender×Relationship | −0.32 | 0.38 | −0.86 | 0.391 |
| Target character: Receiver-Sender×Relationship | −0.05 | 0.37 | −0.14 | 0.888 |
| Target character: Target-Sender×Age | 0.02 | 0.37 | −0.05 | 0.959 |
| Target character: Receiver-Sender×Age | −0.16 | 0.37 | −0.45 | 0.655 |
| Gender×Relationship | 0.24 | 0.31 | 0.08 | 0.940 |
| Gender×Reputation | 0.22 | 0.32 | 0.69 | 0.492 |
| Gender×Age | 0.22 | 0.32 | 0.68 | 0.497 |
| Relationship×Reputation | 0.12 | 0.31 | 0.40 | 0.693 |
| Relationship×Age | −0.49 | 0.31 | −1.60 | 0.110 |
| Reputation×Age | 0.24 | 0.31 | 0.78 | 0.434 |

*Note.* Significance：***:＜ .001; **:＜ .01; *:＜ .05.
